# Data on sensory profile of green Spanish-style table olives studied by Quantitative Descriptive Analysis

**DOI:** 10.1016/j.dib.2018.08.075

**Published:** 2018-08-30

**Authors:** A. López-López, A.H. Sánchez-Gómez, A. Montaño, A. Cortés-Delgado, A. Garrido-Fernández

**Affiliations:** Departamento de Biotecnología de los Alimentos, Instituto de la Grasa (CSIC), Campus Universitario Pablo de Olavide, Edificio 46, Ctra. Utrera km 1, 41013 Sevilla, Spain

**Keywords:** Green table olives, Multivariate analysis, Panel performance, Sensory analysis, Virtual panel

## Abstract

This article contains processed data related to the research published in “Sensory profile of green Spanish-style table olives according to cultivar and origin” [1]. It provides information on the physicochemical characteristics of the analysed samples and the results of the multivariate analysis used in the above-commented paper. Particularly, it includes: i) the values of pH, titratable acidity, combined acidity, and NaCl for batches according to samples, ii) the scores given to each descriptor by the panelists according to samples, iii) the histogram of the overall scores for descriptor, iv) the boxplot of descriptors over samples, v) the effect of samples and contribution of panelists to the interaction sample∙panelist, vi) correlation between the panelists and the whole panel, vii) panelist performance, viii) panel repeatability, ix) sensory profile of samples (spider graph), x) adjusted means for descriptor according to samples, xi) prevalence of descriptors on samples, xii) product effect as assessed by p-value.

**Specifications table**

**Table:** 

Subject area	Biochemistry
More specific subject area	Sensory Analysis
Type of data	Tables, Figures, Text file
How data was acquired	Sensory profiles were acquired by analysis of table olives by a trained panel, using a set of descriptors previously agreed between the panelists and the panel leader. Scores were obtained from an evaluation sheet by reading the marks for each descriptor in an unstructured 1–11 scale.
Data format	Raw and analysed data
Experimental factors	Batches of spontaneous green Spanish-style table olives from diverse origins and cultivars. Therefore, the experimental factors were: cultivar and growing area
Experimental features	The design consisted of 2 batches of Manzanilla (M), Gordal (G), and Hojiblanca (H) from Almendralejo (Am), Casariche (C), Alameda, (Al), Posadas (P), Utrera (U), Estepa (E), Alcalá de Guadarira (AG), and Arahal (A). Treatments are combinations of the appropriate levels of these factors
Data source location	Seville, Spain, 37°21’36.5’’N; 5°56’18.6’’W
Data accessibility	The data are available with this article

**Value of the data**

• The data include the physicochemical characteristics of diverse batches of green Spanish-style table olives prepared from the main cultivars (M, G, and H) from diverse origins, where they were also subjected to processing. The information is important for the characterization of the evaluated samples.
• The data matrix, containing the scores assigned by panelists to each descriptor according to samples, is critical for assessing the possible influences of cultivars and cultivation origins.
• The multivariate data analysis presented here is an ideal tool for the evaluation of the green Spanish-style table olive profiles, according to the origin and cultivar. The data analysis could include ANOVA and multivariate analysis. Results found here encourage the realisation of similar studies with other cultivars and countries.
• As deduced from data, only a few descriptors were enough to describe significant differences among treatments. However, ANOVA and multivariate analysis were useful to properly assess the panel performance and panelists scoring, as well as the discrimination among samples. The methodology may apply to future studies.

## 1 Data

**Fig. 1:**
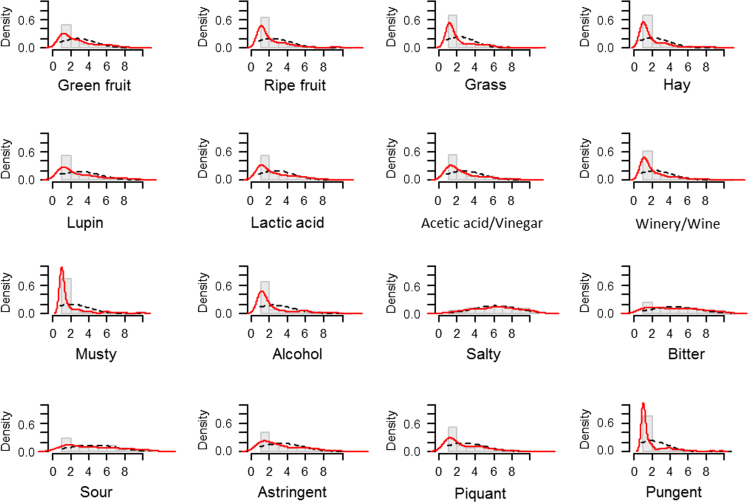
Histogram of the overall scores given by the panelists to the different descriptors.

**Fig. 2:**
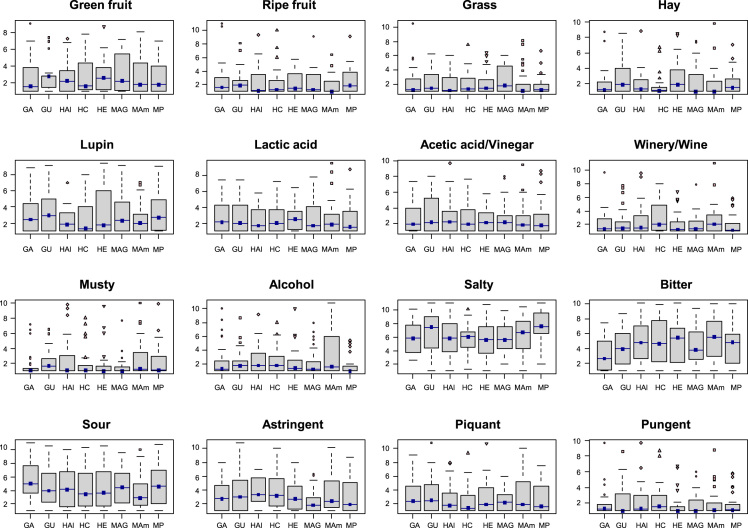
Boxplots of descriptors׳ scores over samples. GA, (cultivar, Gordal; origin, Arahal (Sevilla)); GU, (Gordal; Utrera (Sevilla)); HAI (Hojiblanca; Alameda (Málaga)); HC, (Hojiblanca; Casariche (Sevilla)); HE, (Hojiblanca; Estepa (Sevilla)); MAG, (Manzanilla; Alcalá de Guadaira (Sevilla)); MAm, (Manzanilla; Almendralejo (Badajoz)); MP, (Manzanilla; Posadas (Córdoba)).

**Fig. 3:**
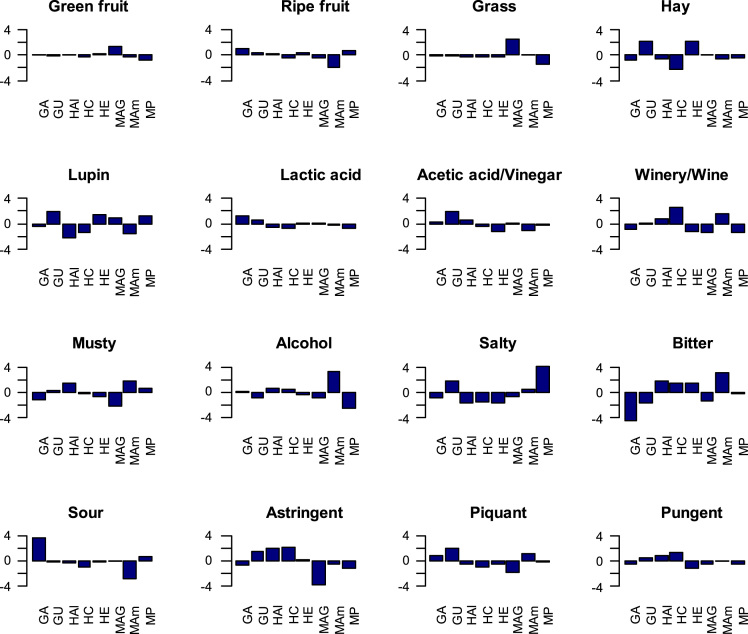
Effect of samples on the interaction sample∙panelist (assessed by their t-values) according to individual descriptors. GA, (cultivar, Gordal; origin, Arahal (Sevilla)); GU, (Gordal; Utrera (Sevilla)); HAI (Hojiblanca; Alameda (Málaga)); HC, (Hojiblanca; Casariche (Sevilla)); HE, (Hojiblanca; Estepa (Sevilla)); MAG, (Manzanilla; Alcalá de Guadaira (Sevilla)); MAm, (Manzanilla; Almendralejo (Badajoz)); MP, (Manzanilla; Posadas (Córdoba)).

**Fig. 4:**
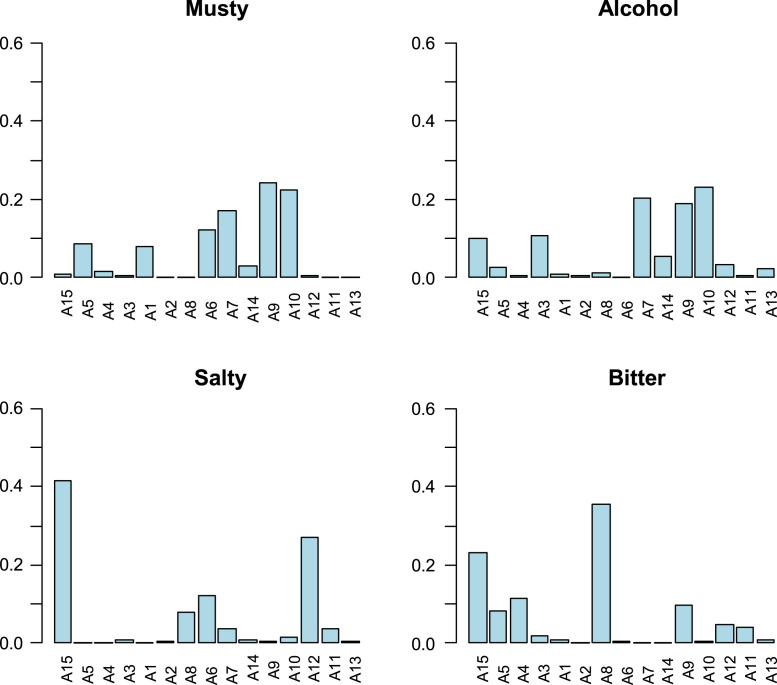
Contributions of panelists to the interaction sample∙panelist (as assessed by the ANOVA coefficients) for musty, alcohol, salty and bitter. Abbreviations for panelists are represented by only an A (assessor) followed by the order number.

**Fig. 5:**
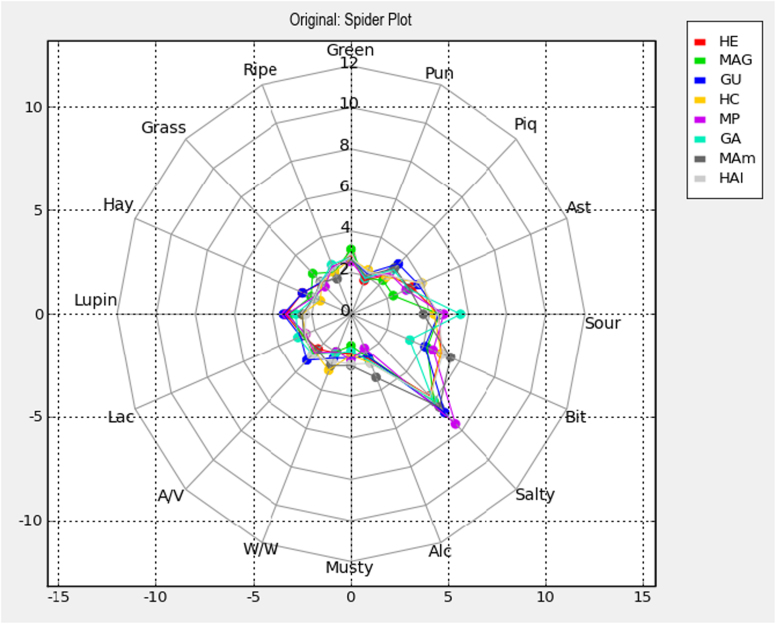
Spider graph showing the sensory profiles (original scores) for the diverse green Spanish-style table olives samples. Green, Green fruit; Pun, pungent; Piq, piquant; Ast, Astringent; Bit, Bitter; Alc, Alcohol; W/W, Winery/Wine; A/V, Acetic acid/Vinegar; Lac, Lactic acid; Ripe, Ripe fruit. HE, (cultivar, Hojiblanca; origin, Estepa (Sevilla)); MAG, (Manzanilla, Alcalá de Guadaira (Sevilla)); GU, (Gordal; Utrera (Sevilla)); HC, (Casariche; Hojiblanca (Sevilla)); MP, (Manzanilla, Posadas (Córdoba)); GA, (Gordal; Arahal (Sevilla)); MAm, (Manzanilla; Almendralejo (Badajoz)); HAI, (Hojiblanca; Alameda (Málaga)).

**Fig. 6:**
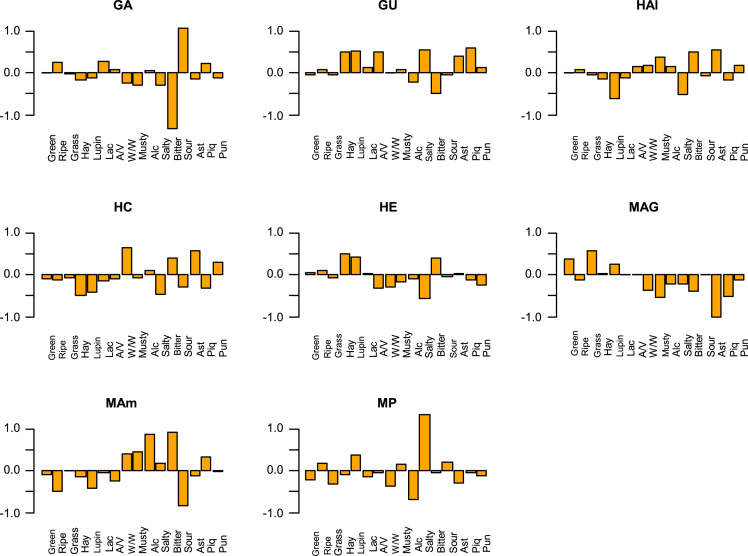
Effect of descriptors on samples, as assessed by their respective coefficients in the ANOVA analysis. Green, Green fruit; Ripe, Ripe fruit; Lac, Lactic acid; A/V, Acetic acid/Vinegar; W/W, Winery/Wine; Alc, Alcohol; Ast, Astringent; Piq, piquant; Pun, pungent. GA, (cultivar, Gordal; origin, Arahal (Sevilla)); GU, (Gordal; Utrera (Sevilla)); HAI, (Hojiblanca; Alameda (Málaga)); HC, (Casariche; Hojiblanca (Sevilla)); HE, ( Hojiblanca; Estepa (Sevilla)); MAG, (Manzanilla, Alcalá de Guadaira (Sevilla)); MAm, (Manzanilla; Almendralejo (Badajoz)); MP, (Manzanilla, Posadas (Córdoba)).

**Fig. 7:**
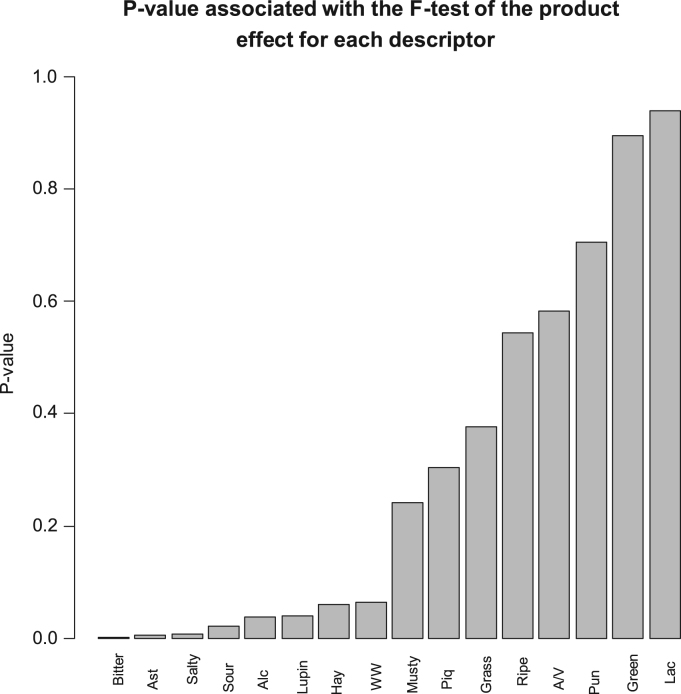
P-values associated to the F-test of the product effect according to descriptors. Low value (p≤0.05) means descriptors with significant differences among samples. Ast, Astringent; Alc, Alcohol; W/W, Winery/Wine; Piq, piquant; Ripe, Ripe fruit; A/V, Acetic acid/Vinegar; Pun, pungent; Green, Green fruit; Lac, Lactic acid.

**Fig. 8:**
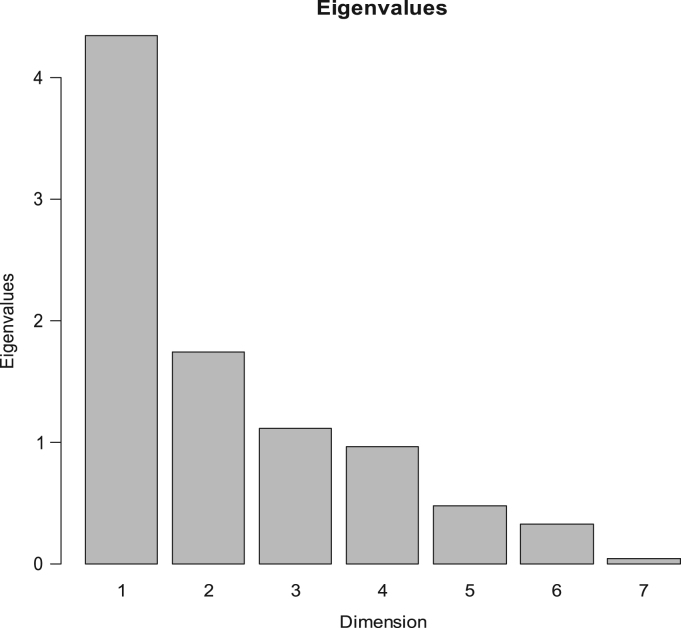
Characterisation of samples. Eigenvalues resulting from the application of PCA to the "condensed" sample averages, deduced from ANOVA analysis to the original data, according to descriptors.

The data cover the physicochemical characteristics of the samples to be analysed (Table 1), the results of the evaluation of the diverse samples by the sensory analysis (Table 2, Fig. 1, Fig. 2, Fig. 3, Fig. 4), the statistical study of them (ANOVA and Multivariate), and the main results with respect to panel and panelists’ performance (Table 3, Table 4, Table 5, Table 6, Fig. 5, Fig. 6), as well as particular aspects related to the discrimination power (Fig. 7, Fig. 8). All these data are related to a previously published research (1).

**Table 1:** Physicochemical characteristics of the studied samples.

**Sample**	**Batch**	**pH**	**Titratable acidity (g lactic acid/L brine)**	**Combined acidity (Eq/L brine)**	**Salt (g NaCl/ L brine)**
GU	1	4.25	6.2	0.092	68
GU	2	4.34	5.2	0.091	67
GA	1	4.05	7.0	0.097	70
GA	2	3.81	8.5	0.096	69
MAG	1	4.09	5.8	0.091	71
MAG	2	4.15	5.2	0.089	67
MP	1	4.32	4.8	0.101	66
MP	2	4.34	4.7	0.101	68
MAm	1	4.65	4.7	0.140	58
MAm	2	4.55	5.2	0.136	57
HC	1	4.27	6.2	0.101	70
HC	2	4.15	6.8	0.100	69
HAl	1	4.28	5.3	0.102	68
HAl	2	4.31	5.2	0.104	69
HE	1	4.07	7.4	0.110	64
HE	2	4.05	7.8	0.108	64

Notes: GU, (cultivar, Gordal; origin. Utrera (Sevilla)); GA, (Gordal; Arahal (Sevilla)); MAG, (Manzanilla, Alcalá de Guadaira (Sevilla)); MP, (Manzanilla, Posadas (Córdoba)); MAm, (Manzanilla; Almendralejo (Badajoz)); HC, (Hojiblanca; Casariche (Sevilla)); HAI, (Hojiblanca; Alameda (Málaga)); HE, (Hojiblanca; Estepa (Sevilla)).

**Table 2:** Scores given by the panelists to the diverse descriptors of the green Spanish-style table olive samples, according to sample, panelist and session.

**Sample**	**Panelist**	**Session**	**Green**	**Ripe**	**Grass**	**Hay**	**Lupin**	**Lac**	**A/V**	**W/W**	**Musty**	**Alc**	**Salty**	**Bit**	**Sour**	**Ast**	**Piq**	**Pun**
HE	A1	S1	2.6	3.0	1.5	2.3	1.4	1.8	1.5	1.0	1.0	1.0	10.8	10.1	5.8	3.4	4.7	1.4
HE	A1	S2	4.3	3.6	1.7	2.0	1.6	2.2	2.1	1.5	1.6	1.4	9.8	9.4	5.0	2.1	2.5	1.4
HE	A2	S1	1.5	1.6	1.6	1.8	1.5	2.6	2.6	1.3	1.4	2.1	7.6	3.6	2.7	1.3	1.0	1.0
HE	A2	S2	1.0	1.0	1.0	1.0	1.0	1.0	1.5	1.0	1.0	1.0	5.5	3.6	2.5	1.0	1.0	1.0
HE	A3	S1	2.6	1.0	1.0	1.0	1.8	3.1	1.0	1.0	1.0	1.0	7.5	6.2	5.7	2.9	1.2	1.0
HE	A3	S2	3.0	2.3	2.1	1.1	1.0	5.6	1.1	1.1	1.0	2.0	6.3	6.3	3.7	3.7	1.4	1.5
HE	A4	S1	4.1	5.2	5.2	4.6	5.0	6.5	1.0	5.5	6.0	6.2	6.4	6.6	6.8	6.8	6.8	6.8
HE	A4	S2	3.2	3.5	2.5	4.8	7.1	5.6	3.4	3.8	6.8	6.0	7.0	6.7	8.3	5.0	4.3	4.3
HE	A5	S1	1.2	4.5	1.0	1.2	1.6	1.0	3.5	2.4	1.0	1.0	5.9	7.0	2.3	1.0	3.8	1.0
HE	A5	S2	1.0	1.0	1.0	1.0	1.0	1.0	4.6	1.0	1.0	3.5	4.7	5.8	4.0	1.0	4.5	1.0
HE	A6	S1	1.6	5.5	3.3	8.5	1.0	2.5	5.3	6.8	9.6	3.9	7.0	7.7	9.0	8.0	2.2	6.5
HE	A6	S2	1.0	1.0	1.0	3.2	8.2	2.6	5.8	3.5	2.4	1.5	9.6	6.5	10.5	5.9	2.0	1.0
HE	A7	S1	6.2	1.0	6.4	1.2	1.0	1.0	1.0	1.0	1.0	1.0	5.5	10.0	9.8	7.6	5.5	1.0
HE	A7	S2	1.3	1.3	1.3	1.3	7.6	1.7	3.7	5.7	1.3	10.0	5.5	9.8	9.2	4.5	10.7	2.0
HE	A8	S1	1.0	1.0	1.0	1.0	1.0	1.0	1.0	1.0	2.3	2.3	5.7	2.0	9.8	2.5	1.0	1.0
HE	A8	S2	1.0	1.0	1.0	1.0	1.5	2.5	1.0	1.0	1.0	1.0	2.8	3.0	8.4	2.0	1.0	1.0
HE	A9	S1	3.8	3.8	3.8	3.8	3.7	2.0	2.1	2.1	2.1	2.2	5.4	1.6	1.7	7.4	2.1	2.1
HE	A9	S2	8.7	2.0	6.0	2.0	6.0	2.9	2.9	2.9	2.9	5.9	2.5	5.7	5.8	2.1	5.7	2.0
HE	A10	S1	3.4	1.0	1.0	8.3	1.0	3.6	1.0	1.0	1.0	1.0	7.1	5.5	1.0	7.2	1.0	1.0
HE	A10	S2	1.0	5.7	1.0	5.3	1.0	1.0	1.0	2.4	1.0	1.0	4.5	1.0	1.0	1.0	1.0	3.6
HE	A11	S1	1.4	1.1	1.5	4.4	1.7	1.2	1.2	1.2	1.2	1.2	3.0	5.4	1.4	4.3	4.7	1.0
HE	A11	S2	1.8	1.0	1.0	1.0	9.4	1.0	1.4	1.2	1.0	1.0	2.7	1.9	1.0	3.7	1.8	1.0
HE	A12	S1	1.2	1.2	1.2	2.4	3.6	3.5	3.4	1.6	1.3	1.3	7.7	5.4	3.6	2.5	2.4	1.5
HE	A12	S2	3.0	1.3	1.4	2.8	6.8	3.4	3.1	1.5	1.3	1.4	8.5	7.0	2.4	3.6	1.3	1.5
HE	A13	S1	2.8	2.7	1.0	1.0	2.3	2.3	2.4	2.5	1.0	2.6	1.8	2.3	2.1	1.0	1.0	1.0
HE	A13	S2	2.2	5.4	1.0	1.0	1.0	4.0	4.0	1.0	1.0	3.8	3.7	1.0	4.8	2.1	2.1	1.0
HE	A14	S1	6.0	1.0	4.8	1.0	6.1	5.3	2.8	1.0	1.0	1.0	1.0	1.0	1.0	1.1	1.0	1.0
HE	A14	S2	3.2	3.2	3.2	3.2	6.1	2.9	2.9	1.8	1.0	2.0	7.8	2.2	2.2	2.2	1.0	1.0
HE	A15	S1	4.3	1.0	1.6	1.0	2.5	1.3	1.0	1.0	1.0	1.0	2.8	1.2	1.2	1.3	1.3	1.0
HE	A15	S2	4.9	4.9	2.6	6.5	5.5	4.0	1.8	1.0	1.0	1.0	3.6	4.0	1.7	3.0	1.3	1.0
MAG	A1	S1	3.6	1.7	2.0	1.6	2.1	2.1	1.5	1.5	1.6	1.4	9.8	9.4	5.0	2.1	2.5	1.4
MAG	A1	S2	2.0	1.9	2.3	1.1	1.1	2.8	1.3	1.4	1.1	1.1	9.2	7.8	4.5	1.2	2.6	1.2
MAG	A2	S1	1.0	1.0	1.8	1.0	1.0	1.0	1.7	1.7	1.1	1.3	4.8	3.8	2.1	1.0	1.0	1.0
MAG	A2	S2	2.2	2.2	1.3	1.3	1.3	2.5	2.3	2.5	1.3	1.3	5.7	5.7	4.1	1.0	1.0	1.0
MAG	A3	S1	2.1	1.0	1.0	1.0	1.0	4.1	1.0	1.0	1.0	1.0	5.2	6.7	5.1	3.6	2.0	1.0
MAG	A3	S2	2.7	1.0	1.0	1.0	1.0	5.0	1.2	2.4	1.0	1.0	7.0	5.1	4.6	1.0	1.0	2.2
MAG	A4	S1	6.2	3.5	4.5	3.2	2.5	6.5	6.0	2.5	2.7	4.9	8.6	6.5	6.5	6.3	4.8	3.9
MAG	A4	S2	5.2	4.0	5.1	3.2	3.8	5.0	5.3	3.0	2.2	6.0	7.2	3.8	6.4	6.1	6.0	3.8
MAG	A5	S1	1.0	1.0	4.6	1.0	4.6	1.0	5.0	1.0	1.0	1.0	8.0	6.7	1.0	1.0	1.0	1.0
MAG	A5	S2	1.7	3.0	1.0	1.0	2.5	1.0	1.0	1.0	3.7	3.3	4.6	4.2	5.5	1.0	1.0	1.0
MAG	A6	S1	1.0	3.7	5.4	3.2	1.0	1.7	2.4	3.5	1.0	1.9	9.9	3.5	8.4	4.7	4.0	6.0
MAG	A6	S2	1.5	4.3	2.7	7.5	5.3	3.7	5.5	3.2	7.7	6.6	3.7	4.9	8.4	3.2	1.0	4.0
MAG	A7	S1	5.9	1.0	5.9	1.0	1.0	1.0	1.0	4.3	1.0	4.3	9.1	7.7	7.0	5.1	4.3	1.0
MAG	A7	S2	1.0	1.0	1.0	3.5	1.0	1.0	1.0	1.7	1.0	5.5	4.7	6.2	7.7	1.7	3.0	1.0
MAG	A8	S1	1.0	1.0	1.0	1.0	1.0	1.0	1.0	1.0	1.0	1.0	6.0	1.2	8.5	1.0	1.0	1.0
MAG	A8	S2	1.0	1.0	1.0	1.0	1.0	1.0	2.0	1.0	1.0	1.0	4.0	8.0	9.5	2.5	1.0	1.0
MAG	A9	S1	3.2	9.1	3.0	6.0	9.1	6.0	8.0	7.9	1.6	8.0	3.0	3.0	8.4	2.9	2.9	2.9
MAG	A9	S2	5.7	1.5	1.8	1.8	7.8	7.8	7.8	2.0	1.5	2.0	5.4	3.3	3.3	2.4	2.4	2.4
MAG	A10	S1	6.8	1.0	5.7	1.0	1.0	1.0	3.0	3.0	1.0	1.0	6.6	1.0	4.1	1.0	4.1	5.4
MAG	A10	S2	6.0	1.0	4.5	1.0	1.0	1.0	1.0	1.0	1.0	1.0	6.8	4.8	1.0	1.0	1.0	3.4
MAG	A11	S1	1.8	1.0	1.0	1.0	8.2	1.0	1.8	1.1	1.1	1.1	2.7	3.3	1.0	1.4	3.3	1.2
MAG	A11	S2	1.1	1.0	1.1	1.0	6.5	1.0	1.5	1.0	1.0	1.0	4.2	4.0	1.6	2.0	2.5	1.0
MAG	A12	S1	3.0	1.8	1.8	7.3	5.0	1.7	3.6	1.3	1.2	1.3	9.3	2.6	4.4	5.4	3.9	1.7
MAG	A12	S2	1.8	1.9	1.8	3.0	3.0	2.6	2.3	1.2	1.2	1.9	3.3	2.6	1.8	1.9	1.1	1.1
MAG	A13	S1	2.3	3.8	1.5	1.0	1.0	1.5	2.7	1.0	1.0	1.0	5.4	2.1	5.5	1.9	4.0	1.0
MAG	A13	S2	1.0	1.0	1.0	1.0	1.0	3.3	2.7	1.0	1.0	1.0	2.0	3.3	2.2	2.5	1.0	1.0
MAG	A14	S1	7.2	1.0	5.8	1.0	7.3	5.7	2.9	1.0	1.0	1.0	7.5	1.0	3.6	1.0	1.0	1.0
MAG	A14	S2	2.7	4.5	4.5	4.5	4.5	4.5	2.5	2.5	1.0	2.4	6.4	1.0	1.0	1.5	1.0	1.0
MAG	A15	S1	6.4	4.0	6.0	3.0	4.6	1.0	1.0	1.0	1.7	1.0	4.3	1.3	1.0	1.3	2.8	1.0
MAG	A15	S2	5.5	1.0	1.8	1.0	3.6	1.4	1.0	1.0	1.0	1.2	5.5	1.5	2.5	1.4	1.0	1.0
GU	A1	S1	6.1	2.4	5.3	2.4	4.1	2.4	1.7	1.9	1.9	1.9	11.0	8.6	4.3	4.8	2.3	3.2
GU	A1	S2	2.4	2.4	3.4	1.9	1.4	2.5	1.2	1.3	1.3	1.3	10.0	8.7	4.5	2.3	2.6	1.2
GU	A2	S1	2.8	1.6	1.2	1.8	1.8	1.7	1.8	1.0	1.0	1.0	8.3	1.9	3.0	1.0	1.0	1.0
GU	A2	S2	2.5	1.0	1.6	1.0	1.0	1.0	1.3	1.5	1.0	1.0	5.9	2.9	2.0	1.0	1.0	1.0
GU	A3	S1	2.8	1.2	3.4	3.4	3.7	4.3	1.5	1.5	1.5	4.7	9.0	6.3	5.1	1.6	1.6	1.6
GU	A3	S2	3.2	1.6	2.4	1.0	1.0	1.0	1.0	1.0	1.0	1.0	8.0	2.9	7.2	1.3	4.8	1.0
GU	A4	S1	2.8	4.1	6.2	3.2	3.3	3.5	7.7	6.7	4.4	5.8	7.4	4.8	6.5	6.0	4.7	3.8
GU	A4	S2	3.1	5.0	3.4	4.0	4.0	5.2	6.0	3.8	4.0	5.1	7.4	4.2	7.1	6.4	5.2	3.7
GU	A5	S1	2.8	4.3	1.0	1.0	3.8	1.0	2.9	1.0	1.0	2.7	6.8	6.3	1.0	1.0	4.7	1.0
GU	A5	S2	1.0	1.0	1.0	1.0	1.0	1.0	5.3	4.0	5.9	2.4	7.0	6.0	2.6	1.0	4.3	1.0
GU	A6	S1	2.8	1.0	3.8	6.3	2.6	6.4	4.8	2.0	6.5	2.4	9.0	4.4	8.3	6.9	8.0	3.5
GU	A6	S2	1.7	3.5	1.6	5.4	7.8	6.5	5.7	7.7	4.7	2.8	8.0	3.8	10.2	4.6	7.8	6.3
GU	A7	S1	2.8	1.0	1.0	4.3	5.0	1.0	5.1	5.1	2.1	8.7	9.5	6.0	7.7	10.8	10.8	1.0
GU	A7	S2	1.0	1.0	1.0	5.3	1.0	1.0	2.6	1.0	2.6	2.6	8.0	8.5	9.0	2.7	1.5	1.0
GU	A8	S1	2.8	1.0	1.0	1.0	1.0	1.0	1.0	1.0	1.9	1.0	5.9	1.0	10.5	9.5	1.0	1.0
GU	A8	S2	1.0	1.0	1.0	1.0	1.0	1.0	1.0	1.0	1.0	2.5	9.0	3.0	6.3	1.0	1.0	1.6
GU	A9	S1	2.8	2.1	5.7	8.5	8.7	7.4	7.4	7.4	2.0	2.0	2.0	6.7	3.4	7.0	2.4	4.8
GU	A9	S2	1.5	8.1	1.2	1.2	8.0	7.3	8.0	1.7	2.9	1.5	1.5	7.5	3.0	5.3	5.2	5.2
GU	A10	S1	2.8	4.8	1.0	1.0	1.7	1.0	1.0	1.0	1.9	1.0	7.8	1.0	1.0	7.6	1.0	8.6
GU	A10	S2	1.0	7.0	1.0	6.7	3.3	1.0	1.0	1.0	3.0	1.0	7.8	1.0	1.0	5.0	1.0	1.0
GU	A11	S1	2.8	1.0	1.4	1.0	7.5	2.0	1.6	1.3	1.7	1.0	4.4	4.1	1.0	1.8	4.5	1.2
GU	A11	S2	1.2	1.1	1.0	1.9	5.3	1.0	1.0	1.0	1.0	1.0	3.7	3.4	4.1	2.0	2.0	1.0
GU	A12	S1	2.8	1.8	1.7	2.0	5.0	3.6	3.8	1.6	1.6	1.7	7.5	5.2	4.3	5.4	4.3	1.7
GU	A12	S2	1.3	2.3	1.2	1.1	2.5	2.5	2.5	1.4	1.7	1.4	3.3	2.3	2.3	3.3	2.4	1.2
GU	A13	S1	2.8	2.0	2.0	1.0	1.0	3.8	5.2	4.2	1.0	1.0	2.8	4.4	3.8	4.0	4.0	1.0
GU	A13	S2	1.0	1.0	1.0	1.0	1.0	2.0	1.0	1.0	1.0	1.0	1.0	1.9	3.3	1.8	1.0	1.0
GU	A14	S1	2.8	2.6	2.6	4.1	4.1	4.4	5.4	2.4	1.0	2.5	10.6	1.0	1.9	1.0	1.0	1.0
GU	A14	S2	7.4	2.3	4.1	2.3	9.1	5.0	5.0	2.2	1.6	2.4	10.0	1.0	1.0	1.0	1.0	1.0
GU	A15	S1	2.8	2.0	1.3	3.6	1.4	1.0	1.0	1.0	1.3	1.0	3.8	2.6	3.7	3.7	5.6	1.0
GU	A15	S2	7.0	1.0	1.5	1.0	1.3	1.0	1.0	1.0	1.0	1.8	6.7	1.0	5.2	1.0	5.0	1.0
HC	A1	S1	1.3	2.6	1.1	1.3	1.4	1.7	1.8	3.4	1.6	1.5	8.2	8.0	4.3	4.4	4.0	1.5
HC	A1	S2	2.6	1.6	2.7	1.1	1.2	2.4	1.1	1.8	1.1	1.1	9.2	9.3	4.0	2.6	2.3	1.6
HC	A2	S1	2.3	1.0	1.8	1.3	1.0	1.0	1.5	1.2	1.0	1.0	4.2	3.1	1.9	1.0	1.0	1.0
HC	A2	S2	1.4	1.4	1.3	1.3	1.3	2.3	2.0	1.8	1.3	1.7	6.3	3.2	2.6	1.0	1.0	1.8
HC	A3	S1	3.0	1.2	2.4	1.1	1.0	3.4	1.1	1.1	1.0	1.0	6.0	7.1	6.5	3.6	1.1	1.0
HC	A3	S2	1.8	2.0	2.0	2.1	1.0	4.1	1.0	2.6	1.0	2.7	6.5	7.3	4.7	4.0	1.2	1.2
HC	A4	S1	4.4	3.0	3.7	2.0	4.3	7.2	7.2	5.4	1.7	6.3	6.0	5.2	6.0	6.0	3.0	2.0
HC	A4	S2	4.5	3.4	4.3	2.7	2.3	5.3	5.4	2.9	2.7	6.2	6.5	6.5	6.5	7.6	6.4	4.0
HC	A5	S1	1.0	1.0	1.0	1.1	1.2	1.1	3.9	1.4	1.0	1.6	4.6	6.4	1.7	3.4	1.0	1.0
HC	A5	S2	1.8	1.0	1.0	1.0	2.0	1.0	4.0	5.7	1.0	1.7	3.9	4.5	1.0	3.9	1.0	1.0
HC	A6	S1	1.0	7.4	1.6	6.2	2.2	3.2	4.9	6.8	5.5	3.4	7.8	4.7	9.5	10.0	3.5	8.7
HC	A6	S2	1.5	6.0	3.8	6.7	4.3	5.4	3.4	4.8	7.2	5.4	6.7	1.1	8.7	5.6	1.0	3.7
HC	A7	S1	1.0	2.7	1.0	1.0	4.7	1.0	3.0	5.8	2.1	5.9	6.0	8.3	9.3	9.3	2.8	8.0
HC	A7	S2	6.0	1.0	3.8	1.0	1.0	1.0	1.0	7.2	1.0	3.0	5.0	7.8	6.7	3.0	1.0	1.6
HC	A8	S1	1.0	1.0	1.0	1.0	1.0	1.0	1.0	1.0	1.3	1.0	3.1	9.2	6.0	1.7	1.0	1.0
HC	A8	S2	1.0	1.0	1.0	1.0	1.0	1.0	1.0	5.0	1.0	1.9	6.2	9.9	6.4	2.5	1.0	1.0
HC	A9	S1	1.9	10.0	1.8	1.8	8.0	3.1	3.1	3.1	5.9	3.1	5.7	7.4	2.6	7.5	2.6	2.6
HC	A9	S2	1.2	1.0	1.1	1.1	1.7	2.3	2.4	8.0	8.0	8.0	6.5	8.5	6.5	2.9	1.0	1.0
HC	A10	S1	6.7	1.0	3.5	1.0	1.0	1.0	1.0	2.1	1.0	1.0	7.4	1.0	1.0	8.1	6.5	1.0
HC	A10	S2	1.0	4.2	1.0	1.0	1.0	1.0	1.9	1.0	2.8	1.0	5.0	1.0	1.0	1.0	1.0	3.7
HC	A11	S1	1.3	1.1	1.0	1.0	6.6	1.1	1.3	1.0	1.0	1.0	2.9	4.0	1.2	1.6	1.6	1.1
HC	A11	S2	1.2	1.5	1.2	3.5	1.0	1.0	1.0	1.0	1.0	2.0	7.3	2.0	1.2	6.3	3.5	2.9
HC	A12	S1	1.2	1.1	1.1	1.1	1.8	1.3	1.2	2.9	1.2	1.3	4.5	2.3	1.4	2.9	3.0	3.0
HC	A12	S2	1.1	1.3	1.2	1.3	1.5	3.4	4.6	1.4	1.6	1.6	6.1	4.6	2.5	3.8	3.2	1.8
HC	A13	S1	1.3	1.0	1.0	1.0	1.0	4.7	2.8	1.0	1.0	3.0	1.3	2.5	5.5	1.5	5.8	4.6
HC	A13	S2	2.2	1.1	2.8	1.0	1.0	1.0	1.0	1.0	1.0	2.3	1.8	2.4	1.8	2.6	1.0	1.0
HC	A14	S1	7.8	1.0	7.5	1.0	7.5	3.7	7.7	1.0	1.0	1.0	8.4	1.0	2.9	1.0	1.0	1.0
HC	A14	S2	7.3	1.5	4.9	1.5	7.1	4.5	1.9	1.9	1.0	1.9	6.2	1.0	1.0	3.0	1.0	1.0
HC	A15	S1	4.7	1.6	1.4	1.3	1.2	1.0	1.0	1.3	1.3	1.0	2.8	1.6	2.7	1.6	2.8	1.0
HC	A15	S2	5.5	1.0	1.0	1.3	4.0	3.8	3.8	4.6	1.0	3.5	10.1	8.6	10.3	4.2	9.3	3.4
MP	A1	S1	2.4	2.2	1.1	1.8	2.2	2.9	1.0	1.0	1.1	1.2	9.0	8.7	3.9	2.0	4.7	1.2
MP	A1	S2	4.2	3.9	2.0	1.0	1.3	3.5	1.1	1.9	1.0	1.0	8.0	9.0	2.6	1.5	1.2	1.1
MP	A2	S1	2.7	1.8	1.2	1.2	1.1	1.6	2.4	1.2	1.3	1.4	7.0	5.8	2.6	1.0	1.0	1.4
MP	A2	S2	2.5	1.8	2.0	1.2	2.4	1.4	1.4	1.9	1.0	1.5	6.5	5.3	3.0	1.0	1.0	1.4
MP	A3	S1	1.3	1.0	1.0	2.0	2.2	4.0	1.5	2.0	1.6	1.0	7.1	5.0	7.0	2.5	1.1	2.1
MP	A3	S2	4.0	4.0	2.0	4.0	4.0	6.3	2.0	2.0	4.0	1.0	5.8	5.8	7.2	1.0	1.0	1.0
MP	A4	S1	5.0	1.9	3.6	3.7	3.7	6.1	7.3	4.9	5.5	5.3	8.1	5.8	7.6	1.0	1.0	1.0
MP	A4	S2	5.0	3.7	5.0	3.0	3.2	4.1	5.7	3.4	2.8	5.0	6.7	1.0	7.0	5.7	6.4	4.1
MP	A5	S1	3.0	1.0	1.1	1.1	5.5	1.0	3.2	2.2	1.0	1.4	6.5	6.7	1.7	1.0	2.1	1.0
MP	A5	S2	1.0	2.6	1.0	1.0	1.0	1.0	4.4	1.0	1.0	2.0	7.3	1.0	5.5	1.0	1.0	1.0
MP	A6	S1	1.3	4.0	1.9	7.0	6.0	4.7	4.0	5.8	2.6	4.5	8.0	5.0	5.8	7.6	4.0	5.8
MP	A6	S2	2.8	5.2	1.8	5.3	7.1	5.7	1.0	2.9	2.9	1.7	9.6	5.9	7.5	7.5	4.7	5.7
MP	A7	S1	1.0	5.4	1.0	4.8	1.0	2.0	1.0	1.0	9.9	1.0	10.0	10.0	9.0	5.5	7.5	1.0
MP	A7	S2	1.0	2.0	1.0	1.0	1.0	1.0	2.5	2.5	4.3	1.0	10.5	7.8	10.4	6.0	2.3	1.0
MP	A8	S1	1.0	1.0	1.0	1.0	1.0	1.0	1.0	1.0	1.0	1.0	7.4	6.0	10.8	1.8	1.0	1.0
MP	A8	S2	1.0	1.0	1.0	1.0	3.5	1.0	1.0	1.0	1.0	1.0	10.0	6.5	6.5	1.0	1.0	1.0
MP	A9	S1	3.7	9.1	3.4	1.6	9.0	8.7	8.7	5.6	6.4	5.4	8.3	4.3	6.8	3.5	4.5	3.4
MP	A9	S2	1.0	1.0	1.0	1.0	1.0	1.0	1.0	1.0	1.0	1.0	11.0	2.0	7.0	5.1	1.0	5.3
MP	A10	S1	1.0	4.9	1.0	1.4	1.0	1.0	1.0	1.0	3.4	1.0	7.6	1.0	1.0	8.7	4.2	1.0
MP	A10	S2	4.5	1.0	3.6	1.0	1.7	1.0	2.5	4.7	3.5	1.0	6.2	1.0	2.0	1.3	1.0	4.0
MP	A11	S1	1.4	1.4	1.2	1.2	5.3	1.1	1.5	1.1	1.1	1.1	4.0	3.4	1.2	1.9	4.6	1.1
MP	A11	S2	1.3	1.0	1.1	1.0	6.0	1.0	1.0	1.0	1.0	1.0	3.5	5.0	1.3	1.6	6.7	1.0
MP	A12	S1	1.1	1.8	1.2	1.3	3.0	2.3	1.8	1.2	1.3	3.8	9.5	4.7	6.2	3.0	3.6	1.3
MP	A12	S2	1.1	3.8	1.5	1.0	4.3	1.0	4.3	1.0	1.1	1.1	7.5	3.5	5.3	5.1	4.5	1.8
MP	A13	S1	1.0	1.0	2.7	2.9	2.0	1.0	1.5	1.0	1.0	1.0	3.9	4.2	1.6	1.4	1.4	1.4
MP	A13	S2	1.0	1.0	1.0	2.1	1.0	1.0	1.7	1.0	1.0	1.0	2.0	3.5	1.8	2.6	1.0	1.0
MP	A14	S1	7.0	1.0	6.7	2.2	8.5	2.2	8.3	1.0	1.0	1.0	9.7	1.0	1.0	1.0	1.0	1.0
MP	A14	S2	5.3	1.9	2.0	1.6	4.9	2.3	2.5	1.6	1.0	2.0	10.0	2.0	2.0	1.0	1.0	1.0
MP	A15	S1	2.2	2.7	1.3	2.6	1.4	1.5	1.1	1.0	1.3	1.0	6.1	1.7	3.5	1.9	1.8	1.2
MP	A15	S2	5.0	1.0	1.0	1.6	3.3	2.4	1.8	1.0	1.0	1.0	9.5	3.4	3.4	6.2	6.2	1.0
GA	A1	S1	1.0	2.6	1.0	1.2	1.4	4.3	1.6	2.8	2.6	1.2	9.6	7.1	3.1	3.5	5.6	1.7
GA	A1	S2	2.0	3.0	1.1	1.0	1.7	3.8	1.0	1.9	1.0	1.0	10.0	6.1	4.1	2.5	1.1	1.0
GA	A2	S1	2.5	2.4	3.5	1.0	1.0	1.0	2.5	2.5	1.0	1.5	4.8	2.3	7.6	1.0	1.0	1.9
GA	A2	S2	1.7	1.2	1.2	1.2	1.0	1.2	2.3	1.1	1.1	1.0	5.7	1.5	6.8	1.0	1.0	1.3
GA	A3	S1	3.0	2.0	1.2	1.2	2.9	5.8	1.7	2.9	1.0	1.7	5.0	4.4	4.8	7.2	1.3	2.2
GA	A3	S2	6.4	3.1	1.6	1.0	1.0	5.3	3.0	3.0	1.0	3.8	3.6	2.0	6.6	1.0	3.0	1.0
GA	A4	S1	3.9	3.0	4.3	2.8	4.7	7.4	7.4	2.1	1.8	6.0	6.7	3.8	6.8	5.7	4.2	3.1
GA	A4	S2	7.0	3.3	5.5	3.7	3.6	3.7	6.2	3.4	2.8	7.0	6.4	1.0	6.4	5.5	6.7	4.3
GA	A5	S1	1.1	1.0	1.3	1.0	4.0	1.0	3.8	1.2	1.0	4.1	3.4	3.3	6.8	1.0	1.0	1.0
GA	A5	S2	5.2	2.0	2.0	2.0	1.3	2.0	4.4	1.5	2.0	2.0	5.6	5.9	5.2	2.0	4.1	2.8
GA	A6	S1	1.5	10.5	3.8	8.7	5.0	7.4	2.9	1.0	6.5	8.4	7.9	2.2	10.7	7.0	4.6	9.7
GA	A6	S2	1.5	5.0	2.3	7.5	6.1	7.3	6.0	4.5	7.2	6.1	6.5	1.8	8.6	8.0	4.0	1.6
GA	A7	S1	9.1	1.2	10.5	1.0	1.0	2.0	1.0	1.0	1.0	10.0	10.1	5.8	11.0	5.5	9.0	5.0
GA	A7	S2	1.0	1.0	1.0	1.0	1.0	2.9	1.1	9.7	1.1	2.5	5.8	7.5	10.5	3.5	2.0	1.0
GA	A8	S1	1.0	1.0	1.0	1.0	1.0	1.0	1.8	1.0	1.0	1.0	5.8	5.9	10.3	1.0	1.0	1.3
GA	A8	S2	1.0	1.0	1.0	1.0	1.0	1.0	6.2	1.0	1.0	1.0	3.3	3.3	10.5	1.0	1.0	1.0
GA	A9	S1	1.0	11.0	1.0	1.0	8.8	4.0	4.0	1.1	1.1	1.3	6.7	3.0	5.2	5.0	4.5	1.2
GA	A9	S2	6.5	1.0	1.0	1.0	1.0	1.0	4.1	1.0	1.0	1.0	8.4	1.0	8.5	1.0	7.2	1.0
GA	A10	S1	1.0	3.1	1.0	1.0	3.2	1.0	1.0	1.0	6.0	1.0	3.7	1.0	4.5	4.1	5.6	1.0
GA	A10	S2	1.0	5.2	1.0	3.6	4.4	1.0	1.0	2.5	1.0	1.0	3.5	1.0	4.5	4.7	1.3	1.3
GA	A11	S1	1.1	1.3	1.2	1.4	4.6	1.1	1.3	1.2	1.2	1.0	4.2	4.3	1.1	3.5	4.6	1.1
GA	A11	S2	1.2	1.3	1.0	1.8	2.8	1.0	1.0	1.0	1.2	1.0	2.6	2.0	2.6	2.5	4.0	1.0
GA	A12	S1	1.1	1.4	1.0	3.0	4.4	2.6	2.0	1.0	1.2	1.2	8.3	5.0	4.0	4.0	2.8	1.3
GA	A12	S2	1.0	1.0	1.0	2.2	2.2	2.3	3.7	1.2	1.3	1.3	5.8	4.5	3.6	4.7	4.0	1.5
GA	A13	S1	2.6	2.7	1.0	1.0	1.4	4.7	1.3	1.5	1.0	1.7	4.3	2.0	4.3	3.0	1.1	1.2
GA	A13	S2	1.0	1.0	1.0	1.0	1.0	1.0	1.0	3.1	1.0	1.8	3.1	1.1	3.0	1.4	1.0	1.8
GA	A14	S1	5.7	1.0	5.7	1.7	5.7	5.7	5.7	1.7	1.0	1.0	8.6	1.0	1.0	1.0	1.0	1.0
GA	A14	S2	3.4	1.8	3.1	1.7	3.0	3.0	1.8	3.4	1.0	1.8	7.0	1.0	1.0	1.0	1.0	1.0
GA	A15	S1	3.8	1.3	1.3	2.9	1.5	1.2	1.2	1.0	1.2	1.0	7.8	5.8	3.8	1.8	1.7	1.0
GA	A15	S2	3.3	1.0	2.7	1.0	2.7	1.2	1.0	1.0	1.0	1.0	3.5	1.0	1.0	1.3	1.3	1.0
MAm	A1	S1	4.4	2.5	3.2	1.0	1.0	2.0	1.2	2.1	3.5	1.0	10.0	10.0	5.0	2.9	2.0	2.0
MAm	A1	S2	2.9	3.0	1.5	1.5	1.0	2.0	2.0	2.8	1.0	1.0	7.7	9.7	2.5	2.3	1.1	1.1
MAm	A2	S1	1.0	1.0	1.0	1.0	1.0	1.6	2.3	1.0	1.0	1.0	8.0	7.2	2.2	1.0	1.0	1.0
MAm	A2	S2	2.3	1.0	1.6	1.0	1.7	1.7	1.7	2.5	1.0	1.9	6.6	4.7	2.9	1.0	1.0	1.1
MAm	A3	S1	4.4	1.0	1.0	3.7	2.3	2.3	1.0	1.0	1.0	6.0	7.4	5.0	1.0	2.6	1.0	1.0
MAm	A3	S2	5.8	1.1	1.0	2.3	1.0	2.3	1.0	1.0	1.0	1.0	3.6	7.7	4.5	5.3	1.0	1.0
MAm	A4	S1	6.0	3.8	4.9	3.4	4.5	4.8	6.3	4.0	3.7	6.4	6.2	5.4	6.5	6.0	5.2	2.9
MAm	A4	S2	5.7	3.5	5.5	4.0	3.5	4.7	6.0	3.4	2.6	4.8	6.7	6.2	6.7	6.3	6.3	4.1
MAm	A5	S1	1.0	1.0	1.0	1.0	2.0	1.0	4.8	3.3	3.6	2.9	8.7	3.0	4.4	1.0	4.8	1.0
MAm	A5	S2	1.0	1.0	1.0	1.0	6.7	1.0	1.0	4.3	1.0	1.0	7.6	5.7	2.8	1.9	1.0	1.0
MAm	A6	S1	4.2	2.0	2.0	6.6	6.1	7.0	4.3	4.9	7.3	6.0	10.1	8.3	6.3	6.9	2.7	8.8
MAm	A6	S2	1.3	6.4	3.9	9.8	3.0	8.4	3.0	4.5	10.0	4.8	5.8	6.5	1.8	2.4	7.0	5.4
MAm	A7	S1	2.0	1.0	1.0	1.0	2.3	1.0	1.0	2.5	1.0	9.0	10.4	9.0	9.0	10.1	10.0	1.0
MAm	A7	S2	1.0	1.0	1.0	1.0	1.0	1.0	3.0	4.5	1.0	8.5	10.0	9.0	10.0	5.8	9.9	2.0
MAm	A8	S1	1.0	1.0	1.0	1.0	3.2	1.0	1.3	1.0	1.0	1.0	9.6	6.0	3.6	1.0	1.0	1.0
MAm	A8	S2	1.0	1.0	1.0	1.0	1.0	1.0	1.0	1.0	4.5	1.0	7.9	7.9	5.7	2.4	1.0	1.0
MAm	A9	S1	4.5	1.8	4.7	1.6	1.6	9.5	9.5	7.8	1.7	8.5	9.8	7.0	4.3	6.6	6.4	1.3
MAm	A9	S2	1.0	4.8	1.0	1.0	1.0	1.0	1.0	11.0	7.5	10.8	5.8	7.4	3.0	5.8	5.7	1.0
MAm	A10	S1	1.0	1.0	7.6	1.0	1.0	4.6	1.0	1.6	1.0	7.9	1.0	1.0	8.5	1.0	1.0	5.0
MAm	A10	S2	4.3	1.0	2.0	1.0	2.2	1.0	1.0	1.0	1.1	1.0	1.7	2.4	1.0	1.5	1.0	5.1
MAm	A11	S1	1.0	1.0	1.0	1.0	7.0	1.1	1.9	1.2	1.1	1.1	3.1	4.7	1.0	1.4	4.8	1.0
MAm	A11	S2	1.1	1.5	1.1	2.5	2.6	2.0	1.1	1.0	1.5	1.1	5.3	7.8	1.3	2.0	5.3	1.2
MAm	A12	S1	1.6	1.2	1.8	1.8	4.4	4.5	4.5	2.7	1.4	1.7	8.3	5.3	4.2	3.0	2.5	1.3
MAm	A12	S2	1.2	2.6	1.2	2.4	2.2	2.3	2.1	1.6	3.2	1.5	4.3	3.7	2.9	2.9	2.4	1.3
MAm	A13	S1	1.0	1.0	1.0	1.0	1.0	1.0	1.5	1.1	2.2	1.0	1.9	2.6	1.4	2.0	1.0	1.0
MAm	A13	S2	1.4	1.0	1.0	1.0	1.4	1.0	1.0	1.0	1.6	1.0	1.9	3.0	1.0	2.8	1.8	1.0
MAm	A14	S1	8.1	1.0	8.1	1.0	3.1	3.1	3.1	3.1	1.0	3.1	7.7	3.1	2.0	2.0	1.0	1.0
MAm	A14	S2	2.0	2.0	2.0	2.0	2.0	2.0	2.0	2.0	6.0	2.0	6.0	3.0	2.0	2.0	1.0	2.0
MAm	A15	S1	2.4	3.0	1.0	1.8	2.0	1.0	1.0	1.0	1.0	1.0	3.8	1.2	1.2	1.2	1.4	1.0
MAm	A15	S2	4.4	1.0	1.0	1.9	2.0	1.2	1.9	1.9	1.0	1.2	4.7	1.7	2.2	3.5	3.2	1.0
HAl	A1	S1	2.5	2.5	1.5	1.3	1.1	2.5	1.0	1.5	1.3	1.3	8.3	9.8	4.9	3.4	6.7	1.8
HAl	A1	S2	3.3	1.3	4.2	1.3	1.4	2.8	1.7	1.1	1.0	1.0	8.8	10.1	4.7	2.8	1.0	1.0
HAl	A2	S1	1.0	1.0	1.0	1.0	1.0	1.0	2.2	1.0	1.0	1.0	4.1	3.4	3.6	1.0	1.0	1.0
HAl	A2	S2	3.0	2.7	2.4	1.5	1.8	1.0	2.2	1.7	1.0	1.5	6.5	4.8	3.0	1.0	1.0	1.3
HAl	A3	S1	3.2	1.2	2.7	3.8	1.0	5.8	2.3	2.3	1.3	3.6	4.8	4.8	6.7	3.2	1.1	1.1
HAl	A3	S2	3.5	2.2	1.0	1.0	3.0	5.0	4.8	3.4	1.0	2.7	5.8	5.4	6.3	3.0	3.1	3.0
HAl	A4	S1	5.8	3.5	4.4	3.6	4.0	4.8	5.6	4.8	3.0	5.7	6.0	6.4	6.3	6.5	7.8	6.5
HAl	A4	S2	5.2	4.0	5.0	4.1	3.3	3.7	5.4	3.3	2.5	5.4	5.8	6.4	5.2	7.0	5.5	3.2
HAl	A5	S1	3.5	1.0	1.9	1.0	4.4	1.0	2.3	1.0	1.0	1.4	5.4	4.2	1.0	5.0	1.0	1.0
HAl	A5	S2	3.0	1.0	1.0	1.0	3.5	1.0	2.0	1.0	1.0	2.0	6.0	3.2	2.0	6.0	2.0	2.0
HAl	A6	S1	1.2	6.5	3.5	8.8	4.3	5.5	7.4	1.7	5.9	4.6	6.4	2.7	8.2	7.0	1.6	4.2
HAl	A6	S2	6.5	3.6	6.0	2.5	1.5	5.7	4.0	4.9	9.8	6.4	2.0	7.0	5.8	5.8	1.3	4.7
HAl	A7	S1	1.0	1.0	1.0	2.2	1.0	1.0	9.7	5.9	4.3	1.7	3.8	7.9	10.0	4.5	3.6	9.7
HAl	A7	S2	4.0	1.0	1.0	1.0	1.0	1.2	3.0	1.0	2.0	2.0	6.0	10.0	9.0	5.8	4.2	2.0
HAl	A8	S1	1.0	1.0	1.0	1.0	1.0	1.0	2.8	1.0	1.0	1.0	1.0	2.0	9.5	1.0	1.0	4.2
HAl	A8	S2	1.0	1.0	1.0	1.0	1.0	2.5	1.0	1.0	1.0	1.0	8.0	9.0	3.0	2.0	1.0	1.0
HAl	A9	S1	2.0	9.3	1.9	1.9	1.9	3.6	3.6	9.0	3.4	3.4	10.2	1.0	6.8	3.0	3.0	3.0
HAl	A9	S2	1.0	9.3	1.0	1.0	1.0	1.0	1.0	9.5	9.3	9.2	9.1	8.0	8.0	4.4	1.7	1.7
HAl	A10	S1	1.0	4.0	1.0	1.0	1.0	1.0	1.0	1.0	8.4	1.0	4.1	3.1	1.0	1.0	5.1	1.0
HAl	A10	S2	1.0	3.5	1.0	2.5	1.8	1.0	2.4	1.0	1.0	1.0	3.2	1.6	1.0	1.7	2.5	1.0
HAl	A11	S1	1.1	1.1	1.1	1.6	2.0	1.1	2.1	1.5	1.1	1.0	4.5	6.7	1.5	7.0	2.9	1.1
HAl	A11	S2	1.3	1.1	1.1	3.5	2.2	1.0	1.0	1.0	1.0	1.0	3.1	5.0	1.3	5.7	4.2	1.3
HAl	A12	S1	1.2	1.9	1.2	3.7	3.7	1.8	1.8	1.9	1.9	1.9	7.2	6.4	5.2	4.8	2.4	1.5
HAl	A12	S2	2.7	1.1	1.1	1.1	3.1	3.1	1.8	1.1	1.1	1.2	3.6	3.4	2.3	3.3	1.2	1.1
HAl	A13	S1	1.0	1.0	1.0	1.0	1.0	1.0	1.0	1.0	1.0	3.8	1.6	1.5	1.5	3.0	1.1	1.2
HAl	A13	S2	1.0	1.0	4.1	1.0	1.0	1.6	1.0	1.0	1.0	4.9	2.8	3.8	1.8	3.7	1.8	1.0
HAl	A14	S1	6.8	1.0	3.0	1.0	2.9	2.9	2.9	2.9	3.0	3.1	8.7	2.7	1.8	3.0	1.0	1.0
HAl	A14	S2	7.3	1.0	5.0	1.5	7.0	5.0	2.0	2.0	1.0	2.0	8.8	1.6	1.6	1.6	1.0	1.0
HAl	A15	S1	4.8	1.0	2.2	1.0	3.0	1.0	1.0	1.0	1.0	1.2	4.4	1.2	1.3	2.3	1.2	1.0
HAl	A15	S2	1.5	1.0	1.0	3.3	3.4	5.6	5.6	4.4	1.0	1.4	11.0	9.5	9.4	6.7	8.0	1.0

Notes: HE, (cultivar, Hojiblanca; origin, Estepa (Sevilla)); MAG, (Manzanilla, Alcalá de Guadaira (Sevilla)); GU, (Gordal; Utrera (Sevilla)); HC, (Hojiblanca; Casariche (Sevilla)); MP, (Manzanilla, Posadas (Córdoba)); GA, (Gordal; Arahal (Sevilla)); MAm, (Manzanilla; Almendralejo (Badajoz)); HAI, (Hojiblanca; Alameda (Málaga)). Abbreviations for panelists are represented by only an A (Assessor) followed by the order number. S1 and S2, first and second session, respectively. Green, Green fruit; Ripe, ripe fruit; Lac, Lactic acid; A/V, acetic/vinegar; W/W, Winery/Wine; Alc, Alcohol; Bit, Bitter; Ast, Astringent; Piq, piquant; Pun, pungent.

**Table 3:** Agreement (correlation) between panelist and the whole panel according descriptor.

	Bit	Hay	W/W	Ast	Sour	Salty	Lupin	Piq	Musty	Grass	Pun	Ripe	Alc	Green	Lac	A/V	Median
A9	0.5428	0.7512	0.6503	0.5277	0.5786	0.1489	0.7704	0.6654	0.6361	-0.0048	0.0610	0.3408	0.7311	0.5360	0.1046	0.4625	0.5394
A7	0.8086	0.5575	0.5896	0.5126	0.4592	0.8368	0.3549	0.5936	0.3769	0.3123	0.6781	0.2813	0.7064	0.4861	0.6223	0.4516	0.5350
A6	0.7267	**-0.3794**	0.5044	0.5245	0.5984	0.5894	0.5458	*0.8637*	0.4378	0.5725	0.2626	0.1702	0.5309	**-0.3144**	0.3201	0.2934	0.5145
A14	0.7302	0.7511	0.2223	0.4889	**-0.7401**	0.6661	0.4889	N/E	0.7241	0.2670	**-0.0269**	**-0.0407**	0.5009	**-0.3867**	0.5153	0.4038	0.4889
A8	0.4109	N/E	0.6911	0.3483	0.8469	*0.8688*	0.0572	N/E	0.4913	N/E	0.4360	N/E	**-0.1668**	**-0.0738**	**-0.1763**	0.1757	0.3796
A10	0.3242	0.8222	**-0.5674**	0.3759	0.1222	0.3163	0.0409	**-0.5097**	0.3470	0.6097	**-0.0382**	0.7067	0.8020	0.5973	**-0.0671**	0.0418	0.3202
A12	0.2299	0.1506	*0.8986*	0.0724	0.3039	0.3736	0.6366	0.3141	0.6881	0.6575	0.5350	**-0.0107**	**-0.5195**	0.7431	0.2942	**-0.3277**	0.3090
A3	0.7977	**-0.1759**	**-0.1006**	0.1893	0.5517	0.3698	0.3016	0.5489	0.2562	**-0.3565**	**-0.0134**	0.7785	0.7399	**-0.2239**	0.0423	0.3498	0.2789
A4	0.8235	0.7459	0.2169	0.3898	0.1197	0.5370	0.2595	**-0.0333**	0.0772	0.3520	**-0.2546**	**-0.0810**	0.3028	0.1716	**-0.0080**	0.5740	0.2382
A13	0.2094	**-0.1131**	**-0.2816**	0.3986	0.4309	0.0042	0.4398	**-0.3004**	0.5373	**-0.2973**	0.5956	0.2433	0.1383	0.3592	0.5584	0.0928	0.2263
A5	**-0.1322**	**-0.1462**	0.7843	0.6310	0.6125	0.6257	**-0.3966**	0.7418	**-0.0514**	0.8740	**-0.0367**	0.4220	**-0.0279**	**-0.1496**	0.7255	0.0222	0.2221
A1	*0.9094*	*0.9306*	0.6498	*0.8783*	**-0.2138**	**-0.1491**	0.7477	**-0.3918**	0.1885	0.0928	0.4542	0.2541	**-0.3679**	**-0.1136**	0.5128	**-0.4015**	0.2213
A11	0.6344	0.0279	**-0.1420**	0.6233	0.3703	0.0806	0.8165	0.3867	0.2818	**-0.4775**	0.7687	**-0.1190**	0.1735	0.1790	0.1670	**-0.0048**	0.1763
A15	0.1462	0.8185	0.7955	0.5149	**-0.0869**	0.3353	0.0437	0.0716	**-0.6919**	*0.8706*	0.6442	**-0.2132**	0.1230	0.6483	**-0.5641**	**-0.0202**	0.1346
A2	0.6325	0.5543	**-0.1546**	0.0300	0.8428	0.6209	0.2441	N/E	**-0.6640**	**-0.0329**	0.0907	0.7259	0.1340	**-0.5229**	**-0.3105**	**-0.3179**	0.0907
Median	0.6325	0.5559	0.5044	0.4889	0.4309	0.3736	0.3549	0.3505	0.3470	0.2897	0.2626	0.2487	0.1735	0.1716	0.1670	0.0928	0.2789

Notes: Abbreviations for panelists are represented by only an A (assessor) followed by the order number. Bit, Bitter; W/W, Winery/Wine; Ast, Astringent; Piq, Piquant; Pun, Pungent; Ripe, Ripe fruit; Alc, Alcohol; Green, Green fruit; Lac, Lactic acid; A/V, Acetic acid/Vinegar. N/E, not estimated by the program. The values in bold and in italic are mentioned as in brown and in blue, respectively, in the article corresponding to reference (1).

**Table 4:** Panelists’ performance. P-values of the F test (by panelist) of the ANOVA model: descriptor score=sample+session.

	Salty	Bit	Sour	Piq	Alc	Hay	W/W	Pun	Ripe	Green	Grass	Lac	Musty	Ast	Lupin	A/V	Median
A3	0.1821	0.2322	0.1618	0.4874	0.7073	0.4370	0.0630	0.8535	0.6144	0.2883	0.1723	0.1840	0.1697	0.7430	0.6184	0.0692	0.2602
A4	0.0726	0.2251	0.1411	0.8878	0.3657	**0.0237**	0.5051	0.5621	0.3006	0.0762	0.9057	0.7066	**0.0142**	0.3423	0.1751	**0.0265**	0.2628
A1	0.1698	**0.0097**	0.2523	0.9637	0.4262	**0.0465**	0.2042	0.7895	0.2688	0.5328	0.1904	**0.0033**	0.8235	0.4047	0.5249	0.7564	0.3368
A9	**0.0102**	0.1583	0.3774	0.2192	0.2166	0.5646	0.1311	**0.0050**	0.9123	0.4788	0.4455	0.5390	0.3030	0.6232	0.3566	0.4321	0.3670
A7	0.0661	0.1648	0.0834	0.4501	0.4704	0.1158	0.8381	0.5010	0.1835	0.8887	0.6830	0.0520	**0.0446**	0.8716	0.6885	0.2998	0.3749
A12	0.8025	0.3717	0.2883	0.3839	0.8048	0.1918	0.3527	0.2480	0.3289	0.5084	0.0987	0.5724	0.4631	0.8850	0.5192	0.9614	0.4235
A14	0.1853	**0.0215**	0.8945	N/E	0.4237	0.6641	0.4588	0.4934	0.7284	0.8481	0.9357	0.2915	0.5713	0.3925	0.4568	0.7492	0.4934
A10	**0.0025**	0.6568	0.3811	0.7035	0.4934	0.0991	0.8137	0.5111	0.2950	0.2845	0.1630	0.5697	0.6492	0.5759	**0.0226**	0.7356	0.5022
A8	0.3627	0.2364	0.3307	N/E	0.6536	N/E	0.4934	0.5383	N/E	0.4934	N/E	0.5741	0.6116	0.6670	0.6078	0.3069	0.5158
A13	0.5644	0.4351	0.4180	0.8123	**0.0003**	**0.0006**	0.5248	0.5423	0.1916	0.5170	0.7487	0.6512	**0.0030**	0.6164	0.6832	0.4561	0.5209
A5	0.0925	0.7669	0.2657	0.1403	0.8933	0.5612	0.3899	0.5612	0.7855	0.5102	0.5543	0.5085	0.6850	**4.3 e-05**	0.7451	0.8548	0.5578
A6	0.6343	0.0814	0.0669	**0.0337**	0.2443	0.8908	0.8451	0.9602	0.4186	0.7001	0.5786	**0.0019**	0.5504	0.3964	0.8511	0.5878	0.5645
A11	0.8488	0.3777	0.7437	0.2974	0.5839	0.7900	0.8073	0.4886	0.6144	0.5472	0.9310	0.5883	0.6387	0.0543	0.6462	0.8727	0.6266
A15	0.7423	0.8129	0.5893	0.5196	0.5979	0.9297	0.5248	0.5060	0.8407	0.6389	0.3730	0.7181	0.7674	0.6981	0.3472	0.6912	0.6651
A2	0.4383	**0.0205**	**0.0012**	N/E	0.9335	0.8867	0.8198	0.2617	0.8106	0.5837	0.8820	0.8755	0.6943	0.4934	0.8614	0.7251	0.7251
Median	0.1853	0.2322	0.2883	0.4687	0.4934	0.4991	0.5051	0.5111	0.5165	0.5170	0.5664	0.5697	0.5713	0.5759	0.6078	0.6912	0.5022

Notes: Abbreviations for panelists are represented by only an A (assessor) followed by the order number. Bit, Bitter; Piq, Piquant; Alc, Alcohol; W/W, Winery/Wine; Pun, Pungent; Ripe, Ripe fruit; Green, Green fruit; Lac, Lactic acid; Ast, Astringent; A/V, Acetic acid/Vinegar. N/E, not estimated by the program. The values in bold are mentioned as in brown in the article corresponding to reference (1).

**Table 5:** Panel repeatability as assessed by the standard deviation of every judge according to descriptor.

	Green	Ripe	Grass	Hay	Lupin	Lac	A/V	W/W	Musty	Alc	Salty	Bit	Sour	Ast	Piq	Pun
A15	1.655	1.459	1.219	1.907	1.023	1.209	1.179	1.070	0.168	0.604	*2.591*	*3.043*	*2.667*	1.727	*2.390*	0.609
A5	1.345	1.466	0.940	0.269	*1.991*	0.255	1.656	1.401	1.571	1.194	1.187	1.932	1.632	0.340	1.386	0.483
A4	0.922	0.710	1.193	0.374	0.977	1.108	0.973	1.016	0.890	0.617	0.570	1.498	0.573	1.335	1.901	1.544
A3	1.036	0.671	0.675	1.162	1.178	1.315	0.681	0.533	0.643	1.791	1.236	1.391	1.377	1.884	0.983	0.768
A1	1.413	0.653	1.092	0.275	0.725	0.291	0.434	0.608	0.673	0.210	0.759	0.638	0.736	0.491	1.528	0.490
A2	0.782	0.684	0.811	0.340	0.488	0.709	0.448	0.617	0.167	0.461	1.314	0.988	0.707	0.075	0.000	0.275
A8	0.450	0.000	0.000	0.000	0.897	0.491	1.263	1.000	1.025	0.573	*2.548*	*2.298*	*2.168*	*2.326*	0.000	0.830
A6	1.681	*2.455*	1.553	*2.606*	*2.579*	0.650	1.782	*2.345*	*2.925*	1.886	*2.148*	1.675	1.627	1.350	1.567	*2.542*
A7	*3.067*	0.905	*2.844*	1.279	*2.302*	0.402	*2.166*	*3.251*	1.464	*3.579*	1.614	1.209	0.982	*2.240*	*3.276*	*2.476*
A14	*2.309*	0.872	1.958	1.155	*2.252*	1.175	1.761	0.739	1.408	0.700	*2.039*	0.503	0.934	0.698	0.000	0.250
A9	*2.413*	*4.294*	1.502	1.769	*2.743*	*2.699*	*2.652*	*2.899*	*2.553*	*3.030*	1.739	*2.174*	*2.202*	1.878	1.794	0.758
A10	*2.128*	1.879	1.695	1.804	0.446	1.032	0.784	1.265	*2.290*	1.725	0.863	1.665	*1.988*	*2.587*	1.503	*2.461*
A12	0.818	0.648	0.226	1.193	1.434	1.024	1.446	0.417	0.525	0.727	1.823	1.363	0.905	1.340	0.983	0.378
A11	0.457	0.200	0.177	1.283	*2.869*	0.361	0.275	0.118	0.214	0.269	1.468	1.574	0.819	1.335	1.349	0.471
A13	0.698	1.138	1.090	0.200	0.410	1.555	1.216	0.992	0.150	0.446	1.265	1.063	1.454	0.941	1.560	0.922

Notes: Abbreviations for panelists are represented by only an A (assessor) followed by the order number. Green, Green fruit; Ripe, Ripe fruit; Lac, Lactic acid; A/V, Acetic acid/Vinegar; W/W, Winery/Wine; Alc, Alcohol; Bit, Bitter; Ast, Astringent; Piq, Piquant; Pun, Pungent. The values in italic are mentioned as in blue in the article corresponding to reference (1).

**Table 6:** Adjusted means for samples (treatments) as deduced by the application of the full model ANOVA according to descriptors.

	W/W	Bit	Musty	Alc	Pun	Ast	Piq	Salty	A/V	Grass	Green	Hay	Lac	Ripe	Sour	Lupin
MAm	2.727	*5.507*	2.517	*3.341*	1.987	3.220	3.150	6.387	2.450	2.203	2.667	2.043	2.603	**1.840**	**3.697**	2.493
HC	*2.973*	4.983	2.010	2.570	2.307	*3.920*	2.520	5.740	2.600	2.133	2.667	**1.693**	2.500	2.190	4.247	2.510
HAl	2.497	5.087	2.443	2.613	2.187	3.873	2.667	5.700	2.853	2.143	2.747	2.040	2.540	2.393	4.457	**2.310**
MP	1.963	4.533	2.237	**1.780**	1.877	3.047	2.783	*7.543*	2.640	1.880	2.527	2.087	2.493	2.503	4.740	3.287
GU	2.323	4.080	2.150	2.240	2.120	3.727	3.423	6.770	3.183	2.167	2.720	*2.680*	2.783	2.407	4.477	3.447
HE	2.027	4.983	1.907	2.377	1.753	3.373	2.710	5.657	2.370	2.123	2.810	*2.690*	2.670	2.427	4.480	3.333
MAG	1.957	4.200	**1.523**	2.250	1.887	**2.337**	2.307	5.997	2.700	*2.763*	3.120	2.207	2.663	2.197	4.523	3.160
GA	2.077	**3.253**	1.777	2.513	1.877	3.180	3.057	5.923	2.767	2.177	2.753	2.020	2.930	2.580	*5.597*	2.813

Notes: W/W, Winery/Wine; Bit, Bitter; Alc, Alcohol; Pun, Pungent; Ast, Astringent; Piq, Piquant; A/V, Acetic acid/Vinegar; Green, Green fruit; Lac, Lactic acid; Ripe, Ripe fruit. MAm, (cultivar, Manzanilla; origin, Almendralejo (Badajoz)); HC, (Hojiblanca; Casariche (Sevilla)); HAI, (Hojiblanca; Alameda (Málaga)); MP, (Manzanilla, Posadas (Córdoba)); GU, (Gordal; Utrera (Sevilla)); HE, (Hojiblanca; Estepa (Sevilla)); MAG, (Manzanilla, Alcalá de Guadaira (Sevilla)); GA, (Gordal; Arahal (Sevilla)). The values in bold and in italic are mentioned as in brown and in blue, respectively, in the article corresponding to reference (1).

## 2 Experimental design, materials and methods

The olives were harvested from the second half of September to the last week of October 2014. They belong to the Manzanilla (M), Gordal (G), and Hojiblanca (H) cultivars grown in different areas of Andalusia and Extremadura (Spain): *Manzanilla*, Alcalá de Guadaira (Sevilla), Posadas (Córdoba), and Almendralejo (Badajoz); *Gordal*, Utrera (Sevilla) and Arahal (Sevilla); *Hojiblanca*, Alameda (Málaga), Estepa (Sevilla), and Casariche (Sevilla). By combining cultivar and origin, the samples were coded as MAG, MP, MAm, GU, GA, HAl, HE, and HC, respectively. The olive samples were elaborated in duplicate batches as green Spanish-style, using polyethylene containers (5.2 kg olives and 3.4 L brine), in the pilot plant facilities of Instituto de la Grasa. They received similar processing treatments. The fruits were debittered with a lye solution (1.90–2.10 g/L) which penetrated 2/3 of the flesh, followed by one washing with tap water for 11–17 h. After washing, brining was carried out by adding a 11% NaCl solution into the containers. The evolution of the spontaneous fermentation was monitored by controlling the pH, titratable acidity, combined acidity, NaCl, and sugar in the brine [2].

After 5 months, there was no residual sugar left, and the process was considered finished. By then, the physicochemical characteristics (Table 1) were considered normal for the style and cultivar and appropriate for the subsequent sensory analysis. This was accomplished by a panel composed of 9 men and 6 women, recruited because of their major role in the implementation of the Sensory Analysis Method for Table Olives [3] and their high level of training due to participation in the habitual sensory analysis of table olives for decades [4]. The panelists were familiarised with the Quantitative Descriptive Analysis (QDA) techniques by training them for 1 h twice a week for two months. After agreement concerning the descriptors to be used and training on the characteristics of the QDA, the panel was used for table olive evaluation. The green Spanish-style table olive samples were taken directly from the fermentation vessels and presented to panelists in a randomised order, contained in standard glasses [5], and coded with three randomly chosen digits. The panelists were asked to mark the intensity of the different descriptors in their corresponding scales. Between tests, the panelists were provided with tap water to cleanse the palate. The scores of the attributes were measured with the exactitude of one decimal point and the results tabulated.

The data were analysed using the SensoMineR v.1.07 software [6]. The program was designed and programmed in R language [7] and collects classical methods usually applied when analysing sensory data as well as others directly conceived in the developers’ laboratory. SensoMineR provided the results of the analyses of variance (ANOVA) models and numerous easy to interpret graphical outputs also generated a virtual panel, by bootstrapping techniques, for the multivariate analysis and construction of product confidence ellipses. The Spider graphs were produced using Panel Check V1.4.2 software [8].

The data matrix scores consisted of 240 rows (8 samples x 2 sessions x 15 panelists) and 19 columns (sample, panelist, session, and the 16 descriptors) (Table 2). Values were first tested for distribution (Fig. 1) and Boxplot graphics for evaluating central tendency and possible outliers (Fig. 2). After the ANOVA, based on the interaction sample∙panelist, the effect of samples (Fig. 3) and the contribution of panelists (Fig. 4) were evaluated. Also, the correlation between panelists and the whole panel (Table 3) is presented, ending the panel checking with the panelist performance (Table 4), the panel repeatability (Table 5), prevalence of descriptors (Fig. 6), and p values associated to the F-test of the product effect according to descriptors (Fig. 7). A spider graph (Fig. 5), the adjusted means for each descriptor and sample (Table 6), and the eigenvalues resulting from the PCA application to the condensed sample averages complete the samples characterisation (Fig. 8).
